# Vaccine receipt and vaccine card availability among children of the apostolic faith: analysis from the 2010-2011 Zimbabwe demographic and health survey

**DOI:** 10.11604/pamj.2016.24.47.8663

**Published:** 2016-05-11

**Authors:** Jennifer Lara Kriss, James Goodson, Zorodzai Machekanyanga, Messeret Eshetu Shibeshi, Fussum Daniel, Balcha Masresha, Reinhard Kaiser

**Affiliations:** 1 Global Immunization Division, Center for Global Health, Centers for Disease Control and Prevention, Atlanta, GA, USA; 2 InterCountry Support Team East and Southern Africa, Regional Office for Africa, World Health Organization, Harare, Zimbabwe; 3 Immunization and Vaccines Development Programme, Regional Office for Africa, World Health Organization, Brazzaville, Republic of the Congo

**Keywords:** Vaccines, immunization, apostolic faith, vaccine refusal, vaccine hesitancy

## Abstract

**Introduction:**

Vaccine hesitancy and refusal continue to be a global challenge to reaching immunization targets, especially among those in traditional or fundamentalist religions. The apostolic faith in Zimbabwe has been historically associated with objection to most medical interventions, including immunization.

**Methods:**

We conducted a descriptive analysis of socio-demographic characteristics and vaccine coverage among apostolic and non-apostolic adults aged 15-49 years and children aged 12-23 months using the Demographic and Health Survey conducted in Zimbabwe during 2010-2011. We used logistic regression models to estimate associations between the apostolic religion and receipt of all four basic childhood vaccinations in the Expanded Program on Immunization, receipt of no vaccinations, and availability of child vaccination card.

**Results:**

Among children aged 12-23 months, 64% had received all doses of the four basic vaccinations, and 12% had received none of the recommended vaccines. A vaccination card was available for 68% of children. There was no significant association between Apostolic faith and completion of all basic vaccinations (aOR = 0.90, 95% CI: 0.69-1.17), but apostolic children were almost twice as likely to have received no basic vaccinations (aOR = 1.83, 95% CI: 1.22-2.77) than non-Apostolic children, and they were 32% less likely to have a vaccination card that was available and seen by the interviewer (aOR = 0.68, 95% CI: 0.52-0.89).

**Conclusion:**

Disparities in childhood vaccination coverage and availability of vaccination cards persist for apostolic in Zimbabwe. Continued collaboration with apostolic leaders and additional research to better understand vaccine hesitancy and refine interventions and messaging strategies are needed.

## Introduction

Vaccination is one of the greatest public health achievements, but vaccine hesitancy and vaccine refusal in both developed and developing countries continue to be a global challenge to reaching immunization targets. The Expanded Programme on Immunization (EPI), launched in 1974, provides free routine vaccinations to children throughout the world [1]. In 2012, the World Health Organization (WHO) Global Vaccine Action Plan set a goal for 90% national coverage with all vaccines in national programs by 2020 [2]. However, WHO estimates that 1.5 million children under age 5 die every year from vaccine-preventable diseases [3]. Individuals question or refuse vaccines based mainly on sociocultural, political, economic, or religious factors, and research is needed to better understand these barriers to vaccination [4–8]. The Apostolic faith has historically objected to most medical interventions, including vaccination. The Apostolic church was created in eastern Zimbabwe in the 1930s based on Christian and traditional African cultural-religious practices [9], and the two main groups today are the Marange and Madhidha groups [10]. The Apostolic church has spread beyond Zimbabwe's borders to several other countries, including neighboring Botswana, Malawi, South Africa, and Zambia [11]. An estimated 5 million apostolic faith members are in the east and southern African sub-region [11], and 3.5 million are in Zimbabwe [10]. The church believes that sickness is the will of God. Therefore, the use of modern medicine is seen as “exhibiting little faith in God” and is strongly discouraged [10–12]. The Apostolic faith emphasizes faith-based healing through prayer and the use of holy water and Apostolic healing concoctions. Many Apostolic parents refuse to have their children immunized against vaccine-preventable diseases or receive medical treatments for illnesses like measles and malaria, which can be life-threatening if left untreated [9]. Doctrine and acceptance of health services vary by Apostolic sub-group [12]. Polygamy is practiced as part of the Apostolic faith, and girls are encouraged to marry as soon as they finish high school, and in some cases after finishing grade seven [9]. The Apostolic faith is characterized by a paternalistic structure of asymmetric power where husbands have most of the decision-making power and wives are essentially treated as minors [10, 13, 14]. Wives have little involvement in any decision-making within the family, including about their own health and that of their children [9, 10]. Members of the apostolic faith on average are more disadvantaged socioeconomically than non-Apostolics in Zimbabwe, with less wealth and education, and are more likely to live in rural areas [10]. For this study, we compared 1) socio-demographic characteristics of members of the apostolic faith with those of the non-Apostolic population in Zimbabwe, and 2) vaccination coverage among children of apostolic caregivers with coverage among children of caregivers of other faith groups. This paper adds to the literature an analysis of the association of apostolic religion with several previously unstudied vaccine outcomes, including receipt of all basic childhood vaccinations, receipt of no vaccinations, and availability of a vaccination card.

## Methods

We analyzed data from the Demographic and Health Survey (DHS) conducted in Zimbabwe during 2010-2011, which collected retrospective data from a nationally representative sample of Zimbabweans. For our analysis, we used the men, women, and children datasets. The DHS uses a two-stage cluster sample design in which enumeration areas are selected as sampling units in the first stage, and households are the sampling units for the second stage. The DHS datasets link women to their children to connect information mothers give during the interview to their children. Our analytic sample was limited to adults aged 15-49 years and children aged 12-23 months who participated in the Zimbabwe DHS 2010-2011. We identified members of the Apostolic faith by self-report. In the descriptive analysis, we examined all religious groups, which included Apostolic, Muslim, Pentecostal, Protestant, Roman Catholic, other Christian, traditional, and no religion. For regression models, we grouped all persons of non-Apostolic faiths together and compared them with Apostolics. During 2010-2011, children in Zimbabwe received the four basic EPI vaccines according to the following schedule: tuberculosis vaccine (BCG) at birth, oral polio vaccine (OPV) and pentavalent vaccine (containing antigens against diphtheria, tetanus, pertussis, Haemophilus influenza type B, and hepatitis B) at 3, 4, and 5 months of age, and measles-containing vaccine (MCV) at 9 months of age [15].

We had several outcomes of interest for children. These included receipt of the four basic childhood vaccines: BCG, OPV, pentavalent, and MCV. We created variables for receipt of all basic vaccinations and receipt of no vaccinations. Vaccination with all basic vaccines was defined as receipt of one dose each of BCG and MCV, and 3 doses each of OPV and pentavalent vaccine. Receipt of no vaccinations was defined as having received no dose of any of the four basic vaccines. We also examined whether a vaccination card was seen by the interviewer. We coded all vaccination outcomes either on the vaccination card if it was seen by the interviewer or on the mother's report if the vaccination card was unavailable. For logistic regression models, we selected adjustment variables based on associations with the outcomes found in prior research, including urban/rural residence [16, 17], mother's education level [10, 18, 19], maternal literacy [20], maternal age [21], and household wealth [10, 21–25]. We also considered the number of children <5 years old in the household and whether polygamy was practiced in the household. We analyzed data using SAS version 9.4 (SAS Institute, Cary, NC), and reported weighted percentages based on sample weights used in the DHS data sets to make sample data representative of the entire population [26]. We considered differences and odds ratios to be statistically significant at p < 0.05.

## Results

The Zimbabwe DHS 2010-2011 had a household response rate of 96%. Among eligible women and men identified in the interviewed households, the response rate was slightly higher for women (93%) than for men (86%); the primary reason for nonresponse was not finding someone at home despite multiple visits to the household [27]. For descriptive analysis of demographic characteristics, we included men (n = 7,104) and women (n = 9,171) aged 15-49 years, of whom 1,955 and 3,396, respectively, were Apostolic. For analysis of childhood vaccinations and vaccination card availability, we included children aged 12-23 months (n = 1,059), of whom 462 were Apostolic. Demographic characteristics of apostolic men and women were significantly different than those of non-Apostolic men and women for all variables except marital status of men (Table 1). Apostolics lived predominantly in rural areas and in the provinces of Manicaland and Mashonaland Central, in the east and northeast of the country. Apostolics represented a relatively small proportion of the population in the capital city of Harare. Apostolic women were more likely to be married than non-Apostolic women and were also more likely to live in polygamous households than non-Apostolic women. Both Apostolic men and women were more likely to have only a primary education and belong to the lower end of wealth quintiles compared with non-Apostolic men and women. Among children aged 12-23 months, 64% had received all doses of the four basic EPI vaccinations at any time before the survey; 12% had received none of the recommended vaccines (Table 2). Apostolic children were slightly less likely to have received all basic vaccines (61%) compared with non-Apostolic children (68%), but this difference was not statistically significant (p =0.058). Apostolics were significantly more likely to have received none of the recommended vaccines (16%) compared with non-Apostolics (9%) (p =0.002). Among male children, Apostolics were less likely to have received all basic vaccinations (57% vs. 67%, p =0.019), but this difference was not evident among females. Seventy percent of children residing in urban areas had received all basic vaccinations, compared with 62% of those residing in rural areas (p <0.001). In both urban and rural areas, Apostolics were significantly more likely than non-Apostolics to have received no vaccines (urban: 19% and 9%, respectively, p =0.013; rural: 15% and 10%, p =0.033).

**Table 1:** Demographic characteristics of adults aged 15-49 years, by Apostolic faith membership - Zimbabwe, 2010-2011

	Women, n (%)^a^			Men, n (%)		
	Non-Apostolic	Apostolic	*p-value*^b^	Non-Apostolic	Apostolic	*p-value*
Total	5,683 (100)	3,488 (100)		5,141 (100)	1,968 (100)	
Age						
15-24	2,394 (42)	1,392 (40)	*.001*	2,148 (42)	960 (49)	*<.001*
25-34	1,818 (32)	1,164 (33)		1,654 (32)	554 (28)	
35+	1,472 (26)	932 (27)		1,340 (26)	455 (23)	
Marital status						
Married/living together	3,295 (58)	2,408 (69)	*<.001*	2,562 (50)	1,022 (52)	*.100*
Divorced/separated	453 (8)	258 (7)		186 (4)	52 (3)	
Widowed	333 (6)	226 (6)		50 (1)	15 (1)	
Never married	1,602 (28)	596 (17)		2,342 (46)	879 (45)	
Polygamous household^c^						
Yes	306 (10)	335 (14)	*<.001*	93 (4)	69 (7)	*.001*
No	2,808 (90)	1,992 (86)		2,469 (96)	953 (93)	
Residence						
Urban	2,686 (47)	862 (25)	*<.001*	2,104 (41)	517 (26)	*<.001*
Rural	2,997 (53)	2,626 (75)		3,037 (59)	1,451 (74)	
Province						
Manicaland	666 (12)	560 (16)	*<.001*	642 (12)	330 (17)	*<.001*
Mashonaland Central	369 (6)	502 (14)		442 (9)	296 (15)	
Mashonaland East	406 (7)	418 (12)		454 (9)	214 (11)	
Mashonaland West	610 (11)	416 (12)		612 (12)	261 (13)	
Matabeland North	329 (6)	114 (3)		277 (5)	72 (4)	
Matabeland South	322 (6)	145 (4)		249 (5)	103 (5)	
Midlands	749 (13)	374 (11)		651 (13)	234 (12)	
Masvingo	500 (9)	409 (12)		458 (9)	127 (6)	
Harare	1,317 (23)	405 (12)		1,038 (20)	269 (14)	
Bulawayo	413 (7)	145 (4)		320 (6)	62 (3)	
Education						
Less than secondary	1,381 (24)	1,400 (40)	*<.001*	1,037 (20)	526 (27)	*<.001*
Secondary or higher	4,302 (76)	2,088 (60)		4,103 (80)	1,442 (73)	
Wealth quintile						
First (poorest)	780 (14)	767 (22)	*<.001*	702 (14)	372 (19)	*<.001*
Second	727 (13)	867 (25)		775 (15)	441 (22)	
Third	935 (16)	746 (21)		926 (18)	445 (23)	
Fourth	1,396 (25)	676 (19)		1,227 (24)	436 (22)	
Fifth (wealthiest)	1,846 (32)	432 (12)		1,512 (29)	274 (14)	
Religion						
Apostolic sect		3,488 (100)			1,968 (100)	
Muslim	43 (1)			42 (1)		
Pentecostal	1,939 (34)			1,030 (20)		
Protestant	1,539 (27)			991 (19)		
Roman Catholic	773 (14)			712 (14)		
Traditional	57 (1)			280 (5)		
Other Christian	768 (14)			550 (11)		
None	558 (10)			1,526 (30)		
Other	6 (0)			10 (0)		

a All numbers and percentages presented are weighted data

b Chi-square tests compare Apostolics to non-Apostolics

c Base: Married or living together

**Table 2:** Percentage^a^ of children aged 12-23 months who received all or no basic vaccines^b^ and percentage with a vaccination card seen, by demographic characteristics - Zimbabwe, 2010-2011

	All basic vaccinations^c^ (%)			
	Total	Non-Apostolic	Apostolic	*p-value*^d^
Total	64	68	61	*058*
Sex				
Male	63	67	57	*019*
Female	66	68	65	*781*
Residence				
Urban	70	74	62	*349*
Rural	62	64	60	*326*
Province				
Manicaland	46	52	41	*184*
Mashonaland Central	67	65	69	*393*
Mashonaland East	80	91	70	*004*
Mashonaland West	73	69	78	*351*
Matabeland North	66	63	73	*518*
Matabeland South	72	71	75	*757*
Midlands	58	64	50	*142*
Masvingo	56	52	59	*640*
Harare	68	75	54	*167*
Bulawayo	83	83	84	*745*
Mother's education				
Less than secondary	52	53	52	*865*
Secondary or higher	70	73	66	*068*
Polygamous^e^				
No	65	69	61	*115*
Yes	54	57	51	*303*
Wealth quintile				
First (poorest)	55	50	58	*174*
Second	62	67	59	*235*
Third	61	66	54	*048*
Fourth	74	75	73	*946*
Fifth (wealthiest)	73	75	67	*480*

a All percentages presented are weighted data

b According to a vaccination card or the mother's report

c BCG, measles, and 3 doses each of DTP/pentavalent vaccine and polio vaccine

d Chi-square tests compare Apostolics with non-Apostolics

e Base: Married or living together

The percentage of children receiving all basic vaccinations was 80% or higher only in the city of Bulawayo (83%) and the province of Mashonaland East (80%). In Manicaland, 46% of children had received all basic vaccinations, and 27% had received none. Comparing Apostolics to non-Apostolics, there were no significant differences except in Mashonaland East, where Apostolics were significantly less likely to have all basic vaccinations (p =0.004) and were significantly more likely to have received no vaccines than non-Apostolics (p =0.047). A vaccination card was seen by the interviewer for 68% of the children. This was significantly lower for apostolic children (63%) than for non-Apostolic children (72%) (p =0.010). Among children living in rural areas, Apostolic males were significantly less likely to have a vaccination card seen at the time of the survey than non-Apostolic males (66% vs. 74%, p = 0.021). Among all children, 22% of caregivers said they had a vaccination card but the interviewer did not see it, 4% said they had a vaccination card at some time but no longer had it, and 6% never had a vaccination card for the child. Among all children aged 12-23 months, 87% received BCG, 74% had received DTP3, 74% received OPV3, and 79% received measles vaccine (Table 3). For all doses of the four basic EPI vaccines, children from the apostolic faith were significantly less likely to be vaccinated compared with non-Apostolic children, with differences in vaccination coverage ranging from 6% to 9%. The largest difference was for the third dose of DTP/pentavalent vaccine, which 69% of Apostolic children had received, compared with 78% of non-Apostolic children (p =0.002). In unadjusted logistic regression models, Apostolic religion was associated with a lower odds of receipt of all basic vaccinations (OR = 0.78, 95% CI: 0.60-1.01), although the association was non-significant at p < 0.05, and significantly associated with no vaccinations received (OR = 1.84, 95% CI: 1.24-2.73) (Table 4). Apostolic religion was negatively associated with a vaccination card seen during the interview (OR = 0.71, 95% CI: 0.55-0.92). In multivariate analysis that controlled for number of children <5 years old in the household, urban or rural residence, mother's education level, maternal literacy, maternal age, polygamy, and household wealth, estimated associations with Apostolic religion were similar to those found in the unadjusted models (Table 4). There was no significant association between Apostolic faith and completion of all basic vaccinations (aOR = 0.90, 95% CI: 0.69-1.17), but Apostolic children were almost twice as likely to have received no basic vaccinations (aOR = 1.83, 95% CI: 1.22-2.77) than non-Apostolic children, and they were 32% less likely to have a vaccination card that was available and seen by the interviewer (aOR = 0.68, 95% CI: 0.52-0.89). Of note, older maternal age at first birth (age 25+ years compared with age <18 years) and higher level of education (secondary or higher compared with less than secondary) were significantly associated with child's receipt of all basic vaccinations while controlling for faith and other variables (aOR = 2.14, 95% CI: 1.10-4.16 and aOR = 1.54, 95% CI: 1.08-2.20, respectively). In models limited to Apostolic only, older maternal age and higher maternal education had higher receipt of all basic vaccinations, but not significantly so (aOR = 1.59, 95% CI: 0.98-2.60 and aOR = 2.00, 95% CI: 0.61-6.58, respectively).

**Table 3:** Percentage^a^ of children aged 12-23 months who received specific vaccine doses^b^ and percentage with a vaccination card seen, by religion - Zimbabwe, 2010-2011

	n^c^		DTP/pentavalent			Polio				All basic vaccinations^d^	No vaccinations	Vaccination card seen
		BCG	1	2	3	1	2	3	Measles			
Total	1,059	87	85	81	74	86	82	74	79	64	12	68
Religion												
Apostolic	462	84	82	76	69	82	78	70	75	61	16	63
Non-Apostolic	597	90	88	84	78	89	86	77	83	68	9	72
p-value^e^		008	015	<001	002	<001	001	004	002	058	002	010
Muslim	4	100	100	100	100	90	90	90	100	90	0	54
Pentecostal	175	88	85	83	73	89	85	76	80	66	10	72
Protestant	155	86	85	83	80	85	83	77	78	69	13	70
Roman Catholic	63	96	96	92	84	96	92	77	94	75	4	76
Traditional	6	86	86	86	86	86	77	77	86	77	14	77
Other Christian	114	93	94	87	84	93	89	82	84	67	5	73
None	80	89	86	80	71	89	81	74	86	60	11	71

a All percentages presented are weighted data

b According to a vaccination card or the mother's report

c Unweighted

d BCG, measles, and 3 doses each of DTP/pentavalent vaccine and polio vaccine

e Chi-square tests compare Apostolics with non-Apostolics

**Table 4:** Associations between demographic characteristics and vaccine outcomes, among children aged 12-23 months - Zimbabwe, 2010-2011

	All basic vaccinations^a^		No vaccinations		Vaccination card seen	
	OR^b^ (95% CI)	aOR^c^ (95% CI)	OR (95% CI)	aOR (95% CI)	OR (95% CI)	aOR (95% CI)
Religion						
Non-apostolic	Ref	Ref	Ref	Ref	Ref	Ref
Apostolic	0.78 (0.60, 1.01)	0.90 (0.69, 1.17)	1.84 (1.24, 2.73)	1.83 (1.22, 2.77)	0.71 (0.55, 0.92)	0.68 (0.52, 0.89)
Number of children <5 years in household						
1	Ref	Ref	Ref	Ref	Ref	Ref
2	1.05 (0.79, 1.38)	1.15 (0.86, 1.53)	0.89 (0.58, 1.38)	0.87 (0.56, 1.35)	1.16 (0.87, 1.54)	1.16 (0.86, 1.54)
3+	0.67 (0.44, 1.02)	0.81 (0.52, 1.25)	1.17 (0.62, 2.21)	1.08 (0.56, 2.09)	0.93 (0.60, 1.44)	0.94 (0.60, 1.47)
Residence						
Urban	Ref	Ref	Ref	Ref	Ref	Ref
Rural	0.60 (0.44, 0.81)	1.01 (0.65, 1.57)	1.03 (0.66, 1.59)	0.88 (0.46, 1.68)	1.22 (0.92, 1.62)	1.34 (0.88, 2.05)
Mother's education						
Less than secondary	Ref	Ref	Ref	Ref	Ref	Ref
Secondary or higher	2.05 (1.57, 2.69)	1.54 (1.08, 2.20)	0.68 (0.45, 1.01)	0.71 (0.42, 1.21)	1.10 (0.83, 1.45)	0.98 (0.67, 1.43)
Maternal literacy						
Illiterate	Ref	Ref	Ref	Ref	Ref	Ref
Literate^d^	2.04 (1.44, 2.90)	1.22 (0.79, 1.90)	0.72 (0.43, 1.21)	0.93 (0.49, 1.77)	1.33 (0.92, 1.90)	1.46 (0.91, 2.32)
Mother's age at first birth						
Younger than 18	Ref	Ref	Ref	Ref	Ref	Ref
18-24	1.24 (0.94, 1.65)	1.04 (0.77, 1.40)	0.86 (0.56, 1.31)	0.94 (0.60, 1.47)	0.97 (0.72, 1.30)	0.92 (0.68, 1.25)
25+	2.98 (1.57, 5.67)	2.14 (1.10, 4.16)	0.31 (0.09, 1.02)	0.38 (0.11, 1.28)	1.08 (0.62, 1.89)	1.02 (0.57, 1.81)
Polygamous						
No	Ref	Ref	Ref	Ref	Ref	Ref
Yes	0.96 (0.66, 1.41)	1.07 (0.72, 1.59)	1.16 (0.66, 2.04)	1.09 (0.62, 1.94)	0.76 (0.52, 1.11)	0.79 (0.53, 1.15)
Wealth quintile						
First (poorest)	Ref	Ref	Ref	Ref	Ref	Ref
Second	1.45 (0.99, 2.12)	1.28 (0.87, 1.89)	0.81 (0.44, 1.48)	0.87 (0.47, 1.62)	1.03 (0.70, 1.54)	1.01 (0.68, 1.52)
Third	1.35 ( 0.93, 1.97)	1.19 (0.80, 1.76)	0.95 (0.53, 1.71)	1.08 (0.59, 1.97)	1.24 (0.83, 1.86)	1.18 (0.78, 1.79)
Fourth	2.29 (1.55, 3.38)	1.76 (1.09, 2.83)	0.82 (0.46, 1.47)	1.04 (0.50, 2.13)	0.86 (0.59, 1.26)	0.89 (0.55, 1.43)
Fifth (wealthiest)	2.28 (1.49, 3.49)	1.58 (0.88, 2.84)	0.89 (0.48, 1.66)	1.27 (0.54, 3.03)	1.03 (0.68, 1.56)	1.08 (0.60, 1.92)

a BCG, measles, and 3 doses each of DTP/pentavalent vaccine and polio vaccine

b Unadjusted odds ratio comparing odds of having received all basic vaccinations versus not received all basic vaccinations

c Adjusts for all covariates presented in this table

d Attended secondary education or higher or can read a full sentence

OR = Odds ratio; aOR = Adjusted odds ratio; CI = Confidence interval

## Discussion

Our analysis of the Zimbabwe DHS 2010–2011 found that only 64% of children aged 12-23 months had received all doses of the four basic EPI vaccines at any time before the survey. Although apostolic children were not significantly different from non-Apostolic children in receipt of all basic vaccinations, apostolic children were more likely to have received none of the basic vaccinations and were less likely to have a vaccination card present at the time of the survey. These associations did not appear to be explained by differences in socio-demographic characteristics between Apostolics and non-Apostolic such as education, household wealth, and residence in rural areas, since these associations remained in multivariate models that adjusted for these variables. Previous studies have shown that Apostolic faith is a significant risk factor for reduced use of health services in general - including maternal and child health services [10], antenatal and postnatal care services, and skilled birth attendance [21, 28] -and increased infant and perinatal mortality rates [29, 30]. Our findings are similar to those in a study analyzing data from the 2009 Zimbabwe Multiple Indicator Monitoring Survey, which found that children in the Apostolic faith had significantly lower vaccination rates against tuberculosis, measles, and polio (7.5, 9.6 and 8.9 percentage points, respectively) compared with non-Apostolic children, even after controlling for other factors [10]. Membership in the Apostolic faith in Zimbabwe has continued to grow (from 20% of the population in 1994 to 27% in 2009) [10], so underuse of medical services by the Apostolic community affects a large proportion of the country's population and – in the case of infectious diseases - might have important consequences for Zimbabwe's non-Apostolic population as well. There has been evidence of outbreaks of vaccine-preventable diseases, including several measles outbreaks, starting among Apostolics before spreading to other communities [11, 31–33]. More generally, vaccine hesitancy and refusal related to religious beliefs and other factors continue to be barriers to high vaccination coverage and prevention of vaccine-preventable diseases. In 2012, the WHO's Strategic Advisory Group of Experts (SAGE) established a working group to study vaccine hesitancy and its determinants [34, 35]. The SAGE working group identified three categories of influence that are determinants of vaccine hesitancy: 1) vaccine- and vaccination-specific issues such as experience with past vaccination and knowledge of vaccine-preventable diseases, 2) individual and social group influences, and 3) contextual influences, which include religion, culture, socioeconomics, influential leaders, and geographic barriers [5].

In addition to Apostolics in Zimbabwe and southern Africa, several other religious groups, such as the Dutch Reformed Church in the Bible Belt of the Netherlands, the Amish and Christian Scientists in the U.S., Orthodox Jewish communities in Israel and Belgium, and Muslim communities in Pakistan and Nigeria, have also declined vaccinations based on their religious doctrine [6, 36–40]. Experience during local outbreaks has shown that religious leaders and parents become more accepting of vaccination during an outbreak, particularly when the outbreak is recognized by health authorities and communication strategies are implemented [36]. During recent vaccination campaigns in Zimbabwe, communication messages and implementation strategies aiming to improve coverage among the apostolic population were used. During measles immunization campaigns conducted in response to the 2010 measles outbreak, national-level advocacy included engaging religious leaders. The Prime Minister's office and the Parliamentary Portfolio Committee on Health held meetings with leaders of the apostolic community to sensitize them to the campaign and to discuss the importance of immunizing their children [11]. Additionally, Ministry of Health and Child Welfare workers conducted house-to-house visits with apostolic sect members to talk to household heads, influential mothers-in-law, and first wives about the importance of child survival interventions. Following communication and advocacy efforts, the nationwide measles supplemental immunization activities in 2010 had an estimated 97% coverage among apostolic children according to a post-campaign coverage survey [11, 41]. However, the survey also found that among those who were not vaccinated during the campaign, 29% gave religion as the reason for non-vaccination. When religious leaders do not support immunization of their members, some countries have introduced regulations to compel immunization of children. For example, Botswana passed a law making it illegal for a caregiver to withhold children from a government-sanctioned immunization program, and Malawi and Swaziland used law enforcement assistance to immunize children of religious objectors and to hospitalize seriously ill children of religious objectors during a recent measles outbreak [11].

This study has a few limitations. First, the DHS is a cross-sectional survey and does not follow-up respondents over time. Therefore, the findings presented in this paper are associational and should not be interpreted as causal. Second, the vaccine outcomes were based on a vaccination card or a mother's report when the vaccination card was not present. Although recording errors might have occurred, a vaccination card was likely a reasonably accurate measure of vaccine doses received because each dose is recorded on the card at the time it is administered. When a vaccination card was unavailable or unseen, a mother's report of vaccine doses received by her child might be less accurate because of recall errors. Therefore, potential recall bias might have occurred if there were differences in recall accuracy between apostolic mothers and non-Apostolic mothers who self-reported their children's immunizations and did not present a vaccination card. Third, DHS does not capture information about membership in apostolic sub-groups. Doctrine varies by Apostolic sub-groups, with some sub-groups being more accepting of immunizations and other medical interventions, and some strongly opposed to all medical interventions, including vaccinations. In our analysis we treated all apostolic groups as homogeneous and were unable to conduct sub-group analysis on heterogeneity of apostolic doctrine. Additionally, some cells had small unweighted sample sizes resulting in limited power to show statistically significant differences.

## Conclusion

This study provides evidence that associations exist between membership in the apostolic faith and lower childhood vaccination coverage and receipt or maintenance of a child's vaccination card. These disparities remain, despite targeted efforts in recent years to work specifically with influential members of the apostolic community to provide education about the importance of vaccinations and to improve immunization coverage of apostolic children. Continued collaboration with influential apostolic leaders, participation in services specifically targeting apostolic communities, and additional research to better understand vaccine hesitancy and to refine interventions and messaging strategies are needed to improve vaccination coverage in this community.

**Funding**: This project was supported by the U.S. Centers for Disease Control and Prevention (CDC). The research was supported in part by an appointment to the Research Participation Program at the CDC administered by the Oak Ridge Institute for Science and Education through an interagency agreement between the U.S. Department of Energy and CDC.

### What is known about this topic

- Apostolic faith is a risk factor for reduced use of health services including maternal and child health services, antenatal and postnatal care services, and skilled birth attendance;
- Apostolic faith is associated with lower vaccination rates against tuberculosis, measles, and polio.

### What this study adds

- Among apostolic children aged 12-23 months in Zimbabwe, 61% received all doses of the four basic EPI vaccines; 16% received none of the recommended vaccines;
- Apostolic faith is associated with receiving none of the basic vaccinations and reduced likelihood of having a vaccination card; however, children of apostolic faith do not have significantly different completion of all basic vaccinations compared with non-Apostolic children.

## References

[1] WHO: Handbook of Resolutions (1974).

[2] World Health Organization (2013). Global vaccine action plan 2011-2020.

[3] World Health Organization Global Health Observatory (GHO) data. Immunization.

[4] Dube E, Laberge C, Guay M, Bramadat P, Roy R, Bettinger J (2013). Vaccine hesitancy: an overview. Human vaccines & immunotherapeutics.

[5] Larson HJ, Jarrett C, Eckersberger E, Smith DM, Paterson P (2014). Understanding vaccine hesitancy around vaccines and vaccination from a global perspective: a systematic review of published literature, 2007-2012. Vaccine..

[6] Michael CA, Ogbuanu IU, Storms AD, Ohuabunwo CJ, Corkum M, Ashenafi S (2014). An assessment of the reasons for oral poliovirus vaccine refusals in northern Nigeria. The Journal of infectious diseases.

[7] Streefland PH (2001). Public doubts about vaccination safety and resistance against vaccination. Health policy.

[8] Goodson JL, Chu SY, Rota PA, Moss WJ, Featherstone DA, Vijayaraghavan M (2012). Research priorities for global measles and rubella control and eradication. Vaccine..

[9] Mpofu E, Dune TM, Hallfors DD, Mapfumo J, Mutepfa MM, January J (2011). Apostolic faith church organization contexts for health and wellbeing in women and children. Ethnicity & Health.

[10] Ha W, Salama P, Gwavuya S, Kanjala C (2014). Is religion the forgotten variable in maternal and child health? Evidence from Zimbabwe. Social science & medicine.

[11] World Health Organization (2012). Regional Office for Africa. Addressing resistance to immunization. The experience from southern Africa. Immunisation and Vaccine Development Unit.

[12] Maguranyanga B (2011). Collaborating Centre for Operational Research and Evaluation. Apostolic religion, health and utilization of maternal and child health services in Zimbabwe.

[13] Horrell S, Krishnan P (2007). Poverty and productivity in female-headed households in Zimbabwe. The journal of development studies.

[14] Maxwell D (1995). Witches, Prophets and Avenging Spirits: the Second Christian Movement in North-East Zimbabwe. Journal of religion in Africa.

[15] UNICEF, WHO Immunization Summary. A statistical reference containing data through 2011.

[16] Mavimbe JC, Braa J, Bjune G (2005). Assessing immunization data quality from routine reports in Mozambique. BMC public health.

[17] Rainey JJ, Watkins M, Ryman TK, Sandhu P, Bo A, Banerjee K (2011). Reasons related to non-vaccination and under-vaccination of children in low and middle income countries: findings from a systematic review of the published literature, 1999-2009. Vaccine..

[18] Jani JV, De Schacht C, Jani IV, Bjune G (2008). Risk factors for incomplete vaccination and missed opportunity for immunization in rural Mozambique. BMC public health.

[19] Oladokun RE, Lawoyin TO, Adedokun BO (2009). Immunization status and its determinants among children of female traders in Ibadan, South-Western Nigeria. African journal of medicine and medical sciences.

[20] Johri M, Subramanian SV, Sylvestre MP, Dudeja S, Chandra D, Koné GK (2015). Association between maternal health literacy and child vaccination in India: a cross-sectional study. Journal of epidemiology and community health.

[21] UNICEF (2011). Access to health services and religion in Zimbabwe. Preliminary findings.

[22] Antai D (2009). Faith and child survival: the role of religion in childhood immunization in Nigeria. Journal of biosocial science.

[23] Antai D (2012). Gender inequities, relationship power, and childhood immunization uptake in Nigeria: a population-based cross-sectional study. International journal of infectious diseases..

[24] Sanou A, Simboro S, Kouyate B, Dugas M, Graham J, Bibeau G (2009). Assessment of factors associated with complete immunization coverage in children aged 12-23 months: a cross-sectional study in Nouna district, Burkina Faso. BMC international health and human rights..

[25] Sia D, Kobiane JF, Sondo BK, Fournier P (2007). Individual and environmental characteristics associated with immunization of children in rural areas in Burkina Faso: a multi-level analysis. Sante..

[26] USAID Guide to DHS Statistics.

[27] (2012). Zimbabwe Demographic and Health Survey 2010-11. Zimbabwe National Statistics Agency and ICF International, Inc.

[28] Hove I, Siziya S, Katito C, Tshimanga M (1999). Prevalence and associated factors for non-utilisation of postnatal care services: population-based in Kuwadzanaperi-urban area; Zvimba district of Mashonaland West Province, Zimbabwe. African journal of reproductive health.

[29] Gregson S, Zhuwau T, Anderson RM, Chandiwana SK (1999). Apostles and Zionists: the influence of religion on demographic change in rural Zimbabwe. Population studies.

[30] Tachiweyika E, Gombe N, Shambira G, Chadambuka A, Mufuta T, Zizhou S (2011). Determinants of perinatal mortality in Marondera district, Mashonaland East Province of Zimbabwe, 2009: a case control study. The Pan African medical journal..

[31] Pomerai KW, Mudyiradima RF, Gombe NT (2012). Measles outbreak investigation in Zaka, Masvingo Province, Zimbabwe, 2010. BMC research notes..

[32] Shibeshi ME, Masresha BG, Smit SB, Biellik RJ, Nicholson JL, Muitherero C (2014). Measles resurgence in southern Africa: challenges to measles elimination. Vaccine..

[33] Tumwine JK (1989). Measles in Chimanimani Zimbabwe. East African medical journal.

[34] World Health Organization Immunization, vaccines and biologicals. SAGE working group dealing with vaccine hesitancy (March 2012 to November 14).

[35] World Health Organization (2014). Meeting of the Strategic Advisory Group of Experts on immunization, October 2014 -conclusions and recommendations. Weekly epidemiological record.

[36] Grabenstein JD (2013). What the world's religions teach, applied to vaccines and immune globulins. Vaccine..

[37] Murakami H, Kobayashi M, Hachiya M, Khan ZS, Hassan SQ, Sakurada S (2014). Refusal of oral polio vaccine in northwestern Pakistan: a qualitative and quantitative study. Vaccine..

[38] Knol M, Urbanus A, Swart E, Mollema L, Ruijs W, van Binnendijk R (2013). Large ongoing measles outbreak in a religious community in the Netherlands since May 2013. Euro surveillance.

[39] Stein-Zamir C, Zentner G, Abramson N, Shoob H, Aboudy Y, Shulman L (2008). Measles outbreaks affecting children in Jewish ultra-orthodox communities in Jerusalem. Epidemiology and infection.

[40] Tanne JH (2013). Measles outbreak in Texas may be drawing to a close. BMJ..

[41] World Health Organization, UNICEF (2010). Summary of Zimbabwe post measles vaccination and vitamin A supplementation post campaign evaluation.

